# Association between Preoperative Long-Chain Polyunsaturated Fatty Acids and Oxidative Stress Immediately after Total Knee Arthroplasty: A Pilot Study

**DOI:** 10.3390/nu13062093

**Published:** 2021-06-19

**Authors:** Yusuke Kubo, Masae Ikeya, Shuhei Sugiyama, Rie Takachu, Maki Tanaka, Takeshi Sugiura, Kaori Kobori, Makoto Kobori

**Affiliations:** 1 Department of Rehabilitation, Kobori Orthopedic Clinic, 548-2 Nearaichou, Kita-ku, Hamamatsu 433-8108, Japan; sugi.shu.nba42@gmail.com (S.S.); rie41506taka@gmail.com (R.T.); sgtakeshi@gmail.com (T.S.); kobori.kao@rose.ocn.ne.jp (K.K.); kobo-cli.04.10.01@snow.ocn.ne.jp (M.K.); 2 Department of Health and Nutrition Sciences, Tokoha University, 1230, Miyakodachou, Kita-ku, Hamamatsu 431-2102, Japan; ikeya@hm.tokoha-u.ac.jp; 3 Rehabilitation Sciences, Seirei Christopher University, 3453 Mikataharachou, Kita-ku, Hamamatsu 433-8558, Japan; maki-t@seirei.ac.jp

**Keywords:** arachidonic acid, docosahexaenoic acid, eicosapentaenoic acid, inflammation, ischemia–reperfusion injury, muscle atrophy, oxidative stress, polyunsaturated fatty acids, tourniquet use, total knee arthroplasty

## Abstract

Quadriceps muscle atrophy following total knee arthroplasty (TKA) can be caused by tourniquet-induced ischemia–reperfusion (IR) injury, which is often accompanied by oxidative stress and inflammatory responses. *n*-3 long-chain polyunsaturated fatty acids (LCPUFAs), such as eicosapentaenoic acid (EPA) and docosahexaenoic acid (DHA), exert antioxidant and anti-inflammatory effects against IR injury, whereas *n*-6 LCPUFAs, particularly arachidonic acid (AA), exhibit pro-inflammatory effects and promote IR injury. This study aimed to examine whether preoperative serum EPA + DHA levels and the (EPA + DHA)/AA ratio are associated with oxidative stress immediately after TKA. Fourteen eligible patients with knee osteoarthritis scheduled for unilateral TKA participated in this study. The levels of serum EPA, DHA, and AA were measured immediately before surgery. Derivatives of reactive oxygen metabolites (d-ROMs) were used as biomarkers for oxidative stress. The preoperative serum EPA + DHA levels and the (EPA + DHA)/AA ratio were found to be significantly negatively correlated with the serum d-ROM levels at 96 h after surgery, and the rate of increase in serum d-ROM levels between baseline and 96 h postoperatively. This study suggested the preoperative serum EPA + DHA levels and the (EPA + DHA)/AA ratio can be negatively associated with oxidative stress immediately after TKA.

## 1. Introduction

Quadriceps muscle weakness following total knee arthroplasty (TKA) persists throughout the long-term postoperative course [1]. It leads to functional deficits, such as decreased walking speed and endurance, reduced stair negotiation ability, and increased risk of falls [2,3,4]. Long-term quadriceps weakness is mainly associated with quadriceps muscle atrophy in the operated extremity [5], which can be more affected by stress responses to tourniquet-induced ischemia–reperfusion (IR) injury than surgical trauma [6,7]. Taken together, treatment of tourniquet-induced IR injury to reduce quadriceps atrophy is a significant challenge to optimize postoperative recovery.

A tourniquet is used during TKA to produce a bloodless surgical field. However, tourniquet use also induces IR injury, which is often accompanied by oxidative stress (overproduction of inactive antioxidative stress-related proteins and reactive oxygen species—ROS) and inflammatory responses (pro-inflammatory cytokine expression and inflammatory cell infiltration), causing damage to tissues subjected to ischemia and reperfusion [8,9,10]. Some studies have demonstrated that tourniquet-induced IR injury alters muscle protein metabolism, including a reduction in protein synthesis [11], increase in protein degradation [7], and upregulation of genes involved in cell stress pathways [12]. These alterations may subsequently contribute to quadricep muscle atrophy [13].

Long-chain polyunsaturated fatty acids (LCPUFAs) have been demonstrated to play an important role in modulating IR injury in various tissues. *n*-3 LCPUFAs, such as eicosapentaenoic acid (EPA) and docosahexaenoic acid (DHA), exert antioxidant and anti-inflammatory effects against IR injury [14], whereas *n*-6 LCPUFAs, particularly arachidonic acid (AA), exhibit pro-inflammatory effects and promote IR injury [15]. Several studies have demonstrated that dietary *n*-3 LCPUFAs prevent IR injury-induced damage to various tissues, including skeletal muscles [16,17,18,19,20,21]. The molecular mechanism underlying this phenomenon is attributed to the antioxidant and anti-inflammatory properties of *n*-3 LCPUFAs. Moreover, a low dietary *n*-6/*n*-3 LCPUFAs ratio has been shown to reduce cardiac muscle damage induced by IR injury by suppressing increased oxidative stress and inflammatory responses [22]. Intake of *n*-3 LCPUFAs and a low *n*-6/*n*-3 LCPUFAs ratio contribute to reducing tourniquet-induced oxidative stress and inflammatory responses immediately after TKA.

This study investigated the association between preoperative serum EPA + DHA levels and the (EPA + DHA)/AA ratio and oxidative stress immediately after TKA. We hypothesized that the higher the preoperative serum EPA + DHA level and (EPA + DHA)/AA ratio, the lower the increase in oxidative stress immediately after TKA.

## 2. Materials and Methods

### 2.1. Study Design and Participants

Fourteen eligible patients with knee osteoarthritis scheduled for unilateral TKA between October 2017 and June 2019 participated in a single-center prospective, correlational, and hypothesis-generating study at an orthopedic clinic in Japan (Table 1). The inclusion criteria were age of 60–79 years, body mass index of 20–30 kg/m^2^, ability to understand information about the study, and signed informed consent. The exclusion criteria were dementia, serious cardiovascular disease (e.g., requiring warfarin or heparin), uncontrolled diabetes (HbA1c ≥ 7%), rheumatoid arthritis, liver, kidney, and digestive diseases, a recent history of cigarette smoking, and participation in the research on preoperative exercise training before TKA in our clinic. All participants were fully informed about the purpose of this study and the potential risks associated with participation. Written informed consent was obtained from all participants in accordance with the Declaration of Helsinki. This study protocol was approved by the Ethics Committee of the Society of Physical Therapy Science (SPTS2017005).

### 2.2. Perioperative Care

All participants were admitted on the day of surgery and discharged on postoperative day 7 and underwent a tricompartmental uncemented TKA (low-contact-stress implant, LCS Complete; DePuy, Johnson & Johnson Co, New Brunswick, NJ, USA) with a medial parapatellar approach, which was performed by two experienced surgeons.

Before wound closure, 1000 mg tranexamic acid was applied topically to the perisurgical area. A tourniquet (ATS 2000; Zimmer, Dover, OH, USA) was applied to the superior aspect of the thigh and inflated to 300 mmHg. Intraoperative anesthesia was performed using combined general and epidural anesthesia in all the participants. The method of anesthesia was decided by the attending anesthesiologist. General anesthesia was induced intravenously with propofol, remifentanil, fentanyl, and ketamine. Epidural puncture and catheterization were performed in the L3-4 or L5-S1 intervertebral space based on the condition of the patient’s intervertebral space. Intraoperative epidural anesthesia was maintained with 0.25–0.5% ropivacaine. During the first 48 h postoperatively, patient-controlled epidural analgesia using 0.2% ropivacaine was provided, programmed to deliver a 3 mL bolus with a lockout interval of 60 min and a background infusion of 3 mL/h. Postoperative analgesia was provided up to a daily maximum dose of loxoprofen sodium (180 mg/day), celecoxib (400 mg/day), or acetaminophen (3000 mg/day) during hospitalization. When standard pain management was insufficient, intravenous flurbiprofen axetil (50 mg) and diclofenac sodium suppository (50 mg) or intramuscular pentazocine (15 mg) were used as rescue analgesics for moderate or severe pain. A wound drainage system was used and removed 48 h after the surgery. Blood transfusions were not performed. Postoperative rehabilitation was similar to that described in a previous study [23].

### 2.3. Study Measurements

For the measurement of serum EPA, DHA, and AA levels, blood sampling was performed immediately before surgery, after a fasting period of at least 12 h. The measurement of serum LCPUFAs levels was outsourced to Medic (Shizuoka, Japan). Subsequently, free fatty acids extracted from the serum were analyzed by the higher multiple reaction monitoring method using ultra-fast liquid chromatography coupled with tandem mass spectrometry (LCMS-8030, Shimadzu Corporation, Kyoto, Japan). Derivatives of reactive oxygen metabolites (d-ROMs) were used as biomarkers for oxidative stress [24]. Earlier studies have used the d-ROMs as biomarkers of oxidative stress resulting from IR injury [25,26]. For the measurement of serum d-ROM levels, blood sampling was performed before surgery and at 2, 24, and 96 h after surgery because IR injury-induced oxidative stress in the skeletal muscle may reach its peak 24 h after IR [27], while the d-ROM levels 96 h after surgery were measured mainly to assess the recovery from the oxidative stress. The blood samples for d-ROM were centrifuged within 5 min at 6000 rpm for 2 min, and the supernatant was stored at −80 °C until analysis. Serum d-ROM levels were measured using a free radical elective evaluator system composed of a spectrophotometer and a centrifuge (FREE Carrio Duo, Wismerll Co. Ltd., Tokyo, Japan) immediately before surgery and at 2, 24, and 96 h after surgery. The d-ROM test is based on the concept that the serum organic hydroperoxide content reflects the content of free radicals that produce it. Standard test procedures were as follows: 20 μL serum sample was mixed with an acid buffer solution (pH 4.8) in a cuvette, which was then supplemented with 20 μL of the chromogen (N, N-diethyl-para-phenylenediamine). Serum hydroperoxides are converted to alkoxy and peroxy radicals by the Fenton reaction under acidic conditions. These newly produced radicals oxidized the chromogen, leading to the formation of corresponding radical cations, which were then identified by absorbance at 505 nm using a spectrophotometer [28]. The level of d-ROMs is expressed in arbitrary units, named Carratelli units (U.CARR), with 1 U.CARR corresponding to 0.08 mg/100 mL H_2_O_2_. The baseline demographic and clinical characteristics of the study participants, including current medical history, Kellgren–Lawrence grade, tourniquet time, and the total amount of ropivacaine dosage in postoperative epidural anesthesia, were obtained from medical records.

### 2.4. Statistical Methods

Based on the Shapiro–Wilk test, continuous variables were presented as means with standard deviations (SD) or medians with interquartile ranges (IQR). Categorical variables were presented as frequencies and percentages. Differences in serum d-ROM levels over time were analyzed using one-way analysis of variance with repeated measures. The α level of 0.05 was chosen for the determination of significance. If significance was achieved in the assessment of the test, a paired *t*-test was performed to evaluate if there were differences in the mean serum d-ROM levels between blood tests. A Bonferroni post hoc correction for multiple comparisons with a significance level of α = 0.05/number of comparisons was used to adjust the α level to determine significance for the paired *t*-test (α = 0.05/6 = 0.008). The association of preoperative serum EPA + DHA levels and the (EPA + DHA)/AA ratio with the postoperative serum d-ROMs and the rate of increase in serum d-ROMs between the baseline and postoperative testing sessions were assessed using Pearson’s correlation coefficient or Spearman’s rank correlation coefficient, as appropriate. The increased rate (%) was calculated using the formula [(postoperative value − preoperative value)/preoperative value] × 100. The threshold for significance was set at *p* < 0.05. All statistical analyses were conducted using IBM SPSS, version 26.0 (SPSS Inc., Armonk, NY, USA).

## 3. Results

The demographic and clinical characteristics of the study participants are presented in Table 1.

**Figure 1:**
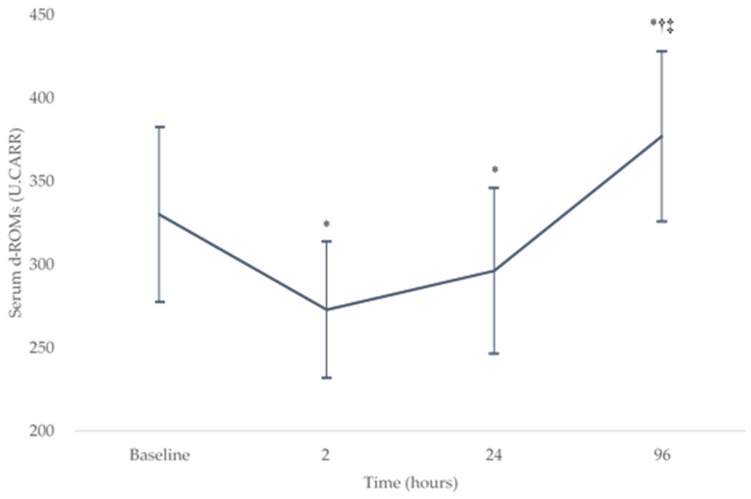
Changes in the serum d-ROMs levels over the baseline and postoperative testing sessions. * *p* < 0.008 compared with the baseline level, † *p* < 0.008 compared with the level at 2 h after surgery, and ‡ *p* < 0.008 compared with the level at 24 h after surgery. d-ROM, derivatives of reactive oxygen metabolites. U.CARR, Carratelli units.

The serum d-ROM levels demonstrated a significant effect in change over the baseline and postoperative periods (*p* < 0.05). Figure 1 shows the change in serum d-ROM levels (mean ± SD) over the baseline (330 ± 53 U.CARR) and postoperative testing sessions (273 ± 41, 296 ± 50, and 377 ± 51 U.CARR at 2, 24, and 96 h after surgery, respectively). The serum d-ROMs decreased significantly from the baseline level at 2 and 24 h after surgery (both *p* < 0.008) and then increased significantly at 96 h after surgery (both *p* < 0.001). There were no significant differences between the serum d-ROMs at 2 and 24 h after surgery (*p* = 0.05). At 96 h after surgery, there was a significant increase in serum d-ROMs compared with the baseline level (*p* = 0.003).

**Figure 2:**
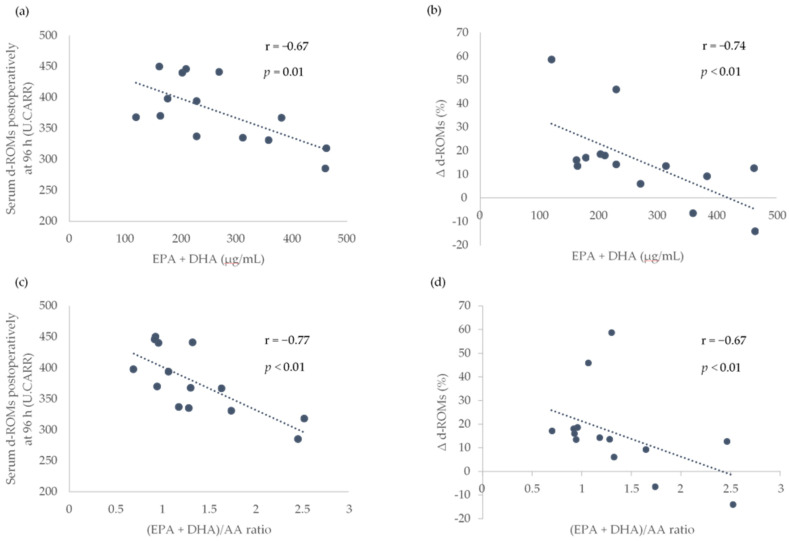
Association of preoperative serum EPA + DHA levels and (EPA + DHA)/AA ratio with the serum d-ROMs postoperatively at 96 h and the rate of increase in serum d-ROMs between the baseline and postoperatively at 96 h (Δ d-ROMs). Scatterplots showing the association between EPA + DHA and d-ROMs postoperatively at 96 h in (**a**), EPA + DHA and Δ d-ROMs in (**b**), EPA + DHA/AA ratio and d-ROMs postoperatively at 96 h in (**c**) and (EPA + DHA)/AA ratio and Δ d-ROMs in (**d**). AA, arachidonic acid; DHA, docosahexaenoic acid; EPA, eicosapentaenoic acid; d-ROMs, derivatives of reactive oxygen metabolites; r, Pearson or Spearman correlation coefficients; U.CARR, Carratelli units. The threshold for significance was *p* < 0.05.

Because a significant increase in the serum d-ROMs between the baseline and postoperative periods was observed at only 96 h after surgery, only the association of the preoperative LCPUFAs with the serum d-ROMs at 96 h after surgery and the rate of increase in serum d-ROMs between baseline and 96 h after surgery (Δ d-ROMs) were analyzed. The preoperative serum EPA + DHA levels and (EPA + DHA)/AA ratio were found to be significantly associated with the serum d-ROMs at 96 h after surgery and Δ d-ROMs (Figure 2).

## 4. Discussion

In the present study, we described the time course of serum d-ROM as a biomarker of oxidative stress before TKA and postoperatively at 2, 24, and 96 h, and showed a negative association of preoperative serum EPA + DHA levels and the (EPA + DHA)/AA ratio with the d-ROMs postoperatively at 96 h and Δ d-ROMs.

Several studies in human models have demonstrated that quadriceps muscle atrophy after TKA can be more affected by stress responses to tourniquet-induced IR injury than surgical trauma [6,7]. IR injury is often accompanied by oxidative stress (ROS overproduction and inactive antioxidative stress-related proteins) and inflammatory responses (pro-inflammatory cytokine expression and inflammatory cell infiltration) [8,9,10]. During IR injury, excessive production of ROS and pro-inflammatory cytokines play important roles in the infiltration of inflammatory cells (leukocytes) into tissues subjected to ischemia and reperfusion, which is characterized by the activation of leukocytes, leukocyte–endothelial cell adhesion, and transmigration. Once inside the interstitium, activated leukocytes release toxic ROS, causing tissue damage [29,30,31]. Carmo-Araújo et al. [27] reported histochemical and morphological alterations induced by 4 h of ischemia and different periods of reperfusion (0, 1, 24, and 72 h) in rat skeletal muscle tissue. After 1 h and 24 h of reperfusion, pronounced inflammatory infiltration and disruption of the myofibril structure were observed. These alterations were progressive from 1 h until 24 h of reperfusion, and a less pronounced inflammatory infiltrate was subsequently observed after 72 h of reperfusion. This study has suggested that ROS produced by activated leukocytes could peak at 24 h of reperfusion, followed by a decrease in the production of ROS.

In the present study, however, serum d-ROMs as a biomarker of oxidative stress decreased from the baseline level at 2 and 24 h after surgery (at 2 and 24 h of reperfusion). This result may be due to the antioxidant and anti-inflammatory effects of the general and epidural anesthetic agents [32,33,34] (used during surgery and until 48 h after surgery, respectively) and deterioration of measurement accuracy in the d-ROMs test. The d-ROM test is a simple assay for analyzing the total amount of hydroperoxides in serum via the Fenton reaction. Reports have indicated that the signal detected in this assay is affected by blood components such as iron ions, which are supplied mostly from Fe^3+^-binding transferrin protein in blood samples [35,36]. During acute phase inflammation, transferrin synthesis in the liver is mainly inhibited by IL-6 [37]. Given that earlier studies of patients undergoing primary TKA indicated that IL-6 reached its peak at 12–24 h, then declined to almost baseline levels after 4–14 days [38,39,40], the serum d-ROM levels at 2 and 24 h after TKA may be underestimated. Taken together, the antioxidant and anti-inflammatory effects of anesthetic agents and deterioration of measurement accuracy in the d-ROMs test due to measurement timing may explain why the serum d-ROM at 2 and 24 h after surgery was lower than that at baseline.

Our findings of a negative association between preoperative serum EPA + DHA levels and the (EPA + DHA)/AA ratio with the d-ROMs postoperatively at 96 h and Δ d-ROMs could be explained by the fact that higher *n*-3 LCPUFAs levels and higher *n*-3/*n*-6 LCPUFAs ratio can attenuate oxidative stress and inflammatory response induced by IR injury. Many studies in animal models have demonstrated that *n*-3 LCPUFAs exhibit antioxidant and anti-inflammatory properties against IR injury [14], whereas *n*-6 LCPUFAs (metabolites of AA) exert pro-inflammatory effects, thereby promoting IR injury [15]. In a rat isolated heart model, *n*-3 LCPUFAs supplementation was associated with lower infarct size compared with the hearts of rats without *n*-3 LCPUFAs supplementation, by reducing ROS production, increasing antioxidant enzyme activities, inhibiting pro-inflammatory signaling pathways (nuclear factor kappa B activity), and attenuating neutrophil (leukocyte) infiltration [16]. Additionally, IR injury-induced tissue damage in the brain, lung, intestine, liver, and skeletal muscle has been ameliorated by *n*-3 LCPUFAs intervention [17,18,19,20,21]. Moreover, a low ratio of dietary *n*-6/*n*-3 LCPUFAs reduces cardiac muscle damage induced by IR injury by suppressing the increase in oxidative stress and inflammatory responses [22]. In the present study, higher preoperative levels of serum EPA + DHA and higher preoperative (EPA + DHA)/AA ratio could contribute to reducing oxidative stress and inflammatory response induced by tourniquet-induced IR injury, resulting in the attenuation of ROS production immediately after surgery. Therefore, we observed a negative association of preoperative serum EPA + DHA levels and the (EPA + DHA)/AA ratio with the d-ROMs postoperatively at 96 h and Δ d-ROMs.

There are several limitations that must be considered. First, the sample size was small, and this study was a correlational study; adjusting for confounding factors, such as age, sex, and tourniquet time, which might affect the association between preoperative LCPUFAs status and postoperative d-ROMs, was not possible. Second, it was not possible to measure factors such as pro-inflammatory cytokines and leukocyte infiltration, which explain the association between preoperative LCPUFAs status and postoperative d-ROMs. Finally, it remains uncertain whether postoperative d-ROMs are associated with muscle atrophy of the lower extremities subjected to IR injury. Future research should pursue a deeper physiological understanding of the association of preoperative LCPUFAs status with d-ROMs immediately after TKA and examine whether suppression of d-ROMs immediately after TKA would optimize long-term muscle volume recovery after surgery.

## 5. Conclusions

We found a negative association between serum EPA + DHA levels and the (EPA + DHA)/AA ratio with the d-ROMs postoperatively at 96 h and Δ d-ROMs. Further research addressing the limitations of this study is required to confirm the validity of our findings.

## Figures and Tables

**Table 1:** Participant demographic and clinical characteristics.

Characteristics	
Participants, *n*	14
Age (years), mean ± SD	71 ± 7
Male, *n* (%)	4 (29)
Body mass index (kg/m^2^), mean ± SD	26 ± 2
EPA + DHA (μg/mL), mean ± SD	268 ± 107
AA (μg/mL), mean ± SD	201 ± 38
(EPA + DHA)/AA ratio, median (IQR)	1.2 (0.9, 1.6)
Current medical history, *n* (%)	
Hypertension	8 (57)
Hyperlipidemia	6 (43)
Diabetes mellitus	1 (7)
Contralateral knee	
OA (KL grade 3 and 4), *n* (%)	9 (64)
TKA, *n* (%)	3 (21)
Tourniquet time (min), mean ± SD	63 ± 5
Postoperative ropivacaine dosage (mL), mean ± SD	148 ± 50

Abbreviations: AA, arachidonic acid; DHA, docosahexaenoic acid; EPA, eicosapentaenoic acid; IQR, interquartile range; KL, Kellgren–Lawrence; OA, osteoarthritis; SD, standard deviation; TKA, total knee arthroplasty.

## Data Availability

The data presented in this study are available upon reasonable request from the corresponding author.
